# Evaluation of Inflammatory Parameters Following Extracorporeal Shock Wave Lithotripsy (ESWL) and Ureteroscopy for the Treatment of Proximal Ureteral Stones

**DOI:** 10.7759/cureus.51882

**Published:** 2024-01-08

**Authors:** Jelena Kovačević Prstojević, Munira Hasanbegović, Jasmin Alić, Verica Mišanović, Almira Lujinović, Azra Metović, Ferid Krupić, Danka Miličić Pokrajac, Admir Hadžimuratović, Lamija Zečević Pašić

**Affiliations:** 1 Urology, Clinical Center University of Sarajevo, Sarajevo, BIH; 2 Pediatric Critical Care, Clinical Center University of Sarajevo, Sarajevo, BIH; 3 Human Anatomy, Faculty of Medicine University of Sarajevo, Sarajevo, BIH; 4 Biology and Human Genetics, Faculty of Medicine University of Sarajevo, Sarajevo, BIH; 5 Anesthesiology, Institute of Clinical Sciences, Sahlgrenska Academy, University of Gothenburg, Gothenburg, SWE; 6 Pediatric Nephrology, Clinical Center University of Sarajevo, Sarajevo, BIH; 7 Clinical Biochemistry and Immunology, Clinical Center University of Sarajevo, Sarajevo, BIH

**Keywords:** kidney stone, inflammation, ureteroscopy, eswl (extracorporeal shockwave lithotripsy), interleukin (il)-6

## Abstract

Introduction

Inflammation can arise as a consequence of both extracorporeal shock wave lithotripsy (ESWL) and ureteroscopy (URS) treatments. Alterations in inflammatory parameters may serve as indicators of kidney injuries and the ensuing inflammation. This study aims to investigate the effects of ESWL and URS procedures on inflammatory parameters for proximal ureteral stone treatment.

Materials and methods

A prospective interventional study comprised 120 patients with confirmed stones measuring less than 10 mm in the upper half of the proximal ureter. These patients were randomly assigned to either the ESWL or URS treatment groups. Laboratory analyses encompassed interleukin-6 (IL-6), leukocyte count, fibrinogen levels, and erythrocyte sedimentation rate (ESR), which were assessed prior to the intervention, on the first postoperative day, and six months later. IL-6 levels in the serum were determined using a chemiluminescence immunoassay (CLIA).

Results

There was no significant difference in IL-6 levels between pre-intervention and the first post-intervention day in patients treated with ESWL (1.8 (1.4-2.59) pg/mL vs. 2.33 (1.22-3.19) pg/mL). However, for patients treated with URS, the pre-intervention IL-6 value was 2.9 (1.9-3.34) pg/mL, and it increased significantly to 7.1 (3.85-28.07) pg/mL on the first post-intervention day (p<0.001). On the first post-intervention day, levels of IL-6, CRP, leukocyte count, and ESR were significantly higher in patients treated with URS compared to ESWL (p<0.001; p<0.001; p=0.03; p=0.03, respectively).

Conclusion

Our research findings suggest that monitoring IL-6 levels can offer valuable insights into the degree of inflammation and tissue damage during and following observed procedures, particularly among patients undergoing URS, even within the initial days post-procedure.

## Introduction

Urolithiasis stands as one of the most prevalent urologic conditions globally, with substantial burden and cost on healthcare systems worldwide. Growing evidence establishes a connection between urolithiasis and various risk factors, encompassing dietary and lifestyle patterns, noncommunicable ailments like diabetes and obesity, and even factors related to global warming [1].

Extracorporeal shock wave lithotripsy (ESWL), widely used for treating urinary stones due to its high effectiveness and non-invasive nature, particularly benefits patients with ureteral stones smaller than 1 cm [2]. However, despite its numerous advantages, ESWL can cause adverse effects such as impaired renal function, perirenal hematomas, hypertension, urinary blockages, and potentially sepsis [3]. It has the potential to harm the delicate renal arteries, leading to temporary hemorrhages, the release of cytokines, and other inflammatory cellular mediators [4].

Over the last few decades, ureteroscopy (URS), a minimally invasive method for extracting kidney and ureteric stones, has gained significant recognition in this field. For multiple reasons, URS has surpassed ESWL in stone surgery [5]. In a ureteroscopic procedure, stone fragments can be removed using a basket or fragmented using a laser. URS is more frequently employed in higher-risk patients, potentially increasing procedural risks and the likelihood of post-procedural infectious complications. This trend is partly due to the high success rates linked to these techniques [6]. The most frequently encountered complications after flexible URS include ureteral strictures, hematuria, infection/sepsis, steinstrasse, pain, and post-voiding symptoms [7].

Currently, there is limited knowledge available in clinical practice for identifying or predicting patients who are more susceptible to developing complications. Importantly, there is a paucity of studies that have investigated the influence of these techniques on inflammatory biomarkers such as interleukin-6 (IL-6) and C-reactive protein (CRP). Clinical and experimental studies have emphasized the pivotal role of IL-6 in renal injury within kidney inflammatory diseases, including acute kidney injury (AKI), among the multitude of implicated inflammatory mediators [8-10]. Acute-phase CRP has been associated with both AKI and chronic kidney disorders (CKD). Nevertheless, the exact role and mechanisms of CRP in AKI and CKD remain largely unexplored [11]. It is worth noting that CRP serves as a valuable biomarker in the diagnosis of urinary tract infections [12].

The objective of the present study was to investigate the effects of ESWL and URS on inflammatory parameters following the treatment of proximal ureteral stones.

## Materials and methods

Patients

This prospective study encompassed 120 patients diagnosed with ureterolithiasis, confirmed to be up to 10 mm in size, located in the upper portion of the proximal third of the ureter. These individuals received treatment at the Clinic of Urology, Clinical Center, University of Sarajevo, in Bosnia and Herzegovina.

Patients were divided into two groups according to their preferred method of treatment. Group A comprised 60 patients diagnosed with ureterolithiasis in the proximal third of the ureter, measuring less than 10 mm, who underwent treatment with ESWL. Group B consisted of 60 patients with ureterolithiasis in the proximal third of the ureter, measuring less than 10 mm, who were treated with URS.

Patients meeting any of the following criteria were excluded from the study: pregnancy, coagulation disorders, uncontrolled urinary tract infections, severe skeletal deformities, significant obesity, arterial aneurysms in close proximity to the treated stone, and other conditions and internal comorbidities contraindicated for ESWL treatment. Urological contraindications for ESWL treatment encompass anatomical abnormalities of the ureter impeding spontaneous elimination of stone fragments, such as ureteral stenosis, impassable distal ureter beyond the stone, disorders of ureteral peristalsis, and compromised kidney function. Anomalies such as ectopia, horseshoe kidneys, and ureter duplications fall under kidney anatomical defects unsuitable for ESWL. General anesthesia is discouraged for URS lithotripsy under certain circumstances. Patients who had a JJ stent placed were not included in the study to avoid potential bias concerning its impact on inflammatory parameters.

**Figure 1:**
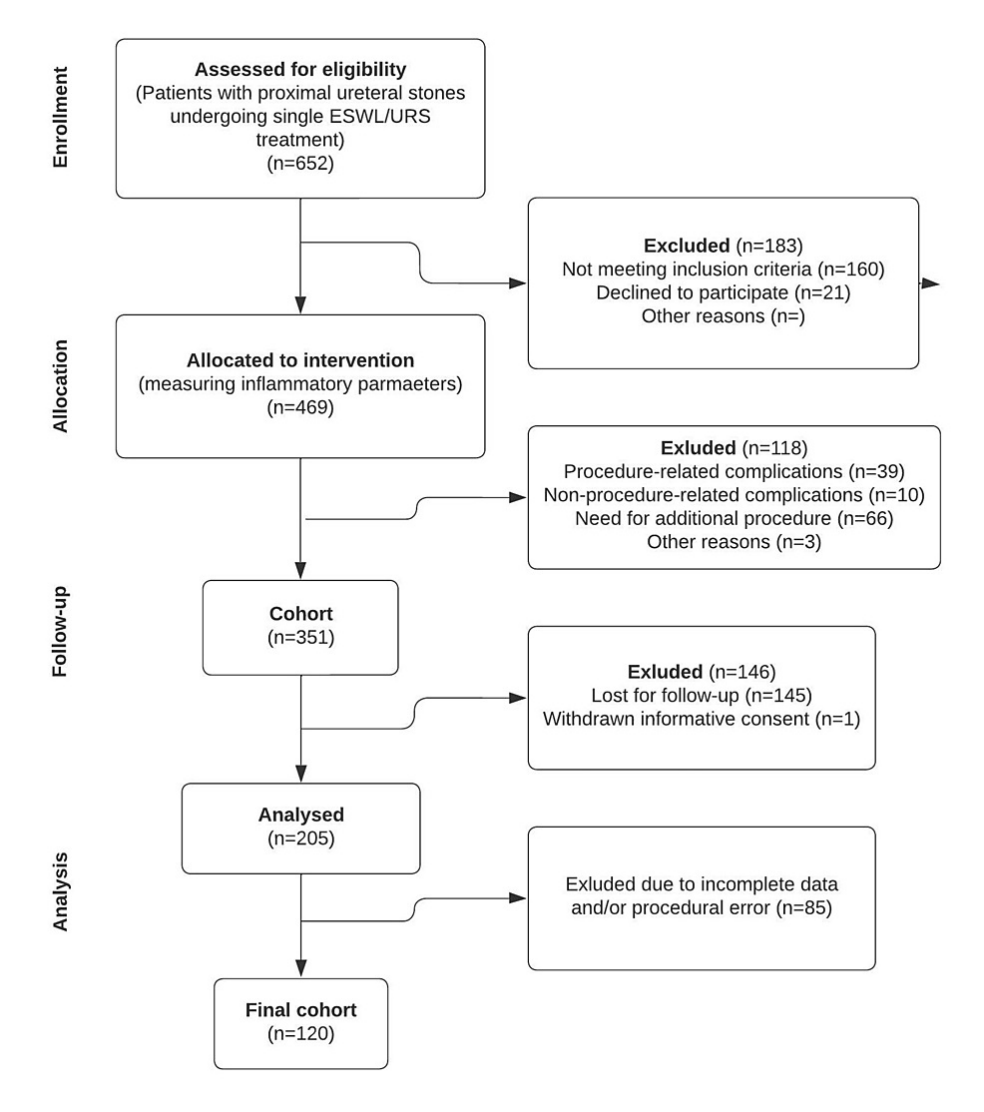
A flow diagram illustrating the progression through the phases of the current trial ESWL: extracorporeal shock wave lithotripsy, URS: ureteroscopy

During the follow-up period, 118 patients were excluded, and 39 of them were due to procedure-related complications (Figure 1). Furthermore, an additional procedure was needed in 66 cases due to obstruction and/or unfavorable stone localization.

Ethical considerations

All procedures involving human patients adhered to the 2013 Helsinki Declaration. The study protocol received approval from the Ethics Committee of Clinical Center University of Sarajevo (approval number: 0302-56349; date: Dec. 30, 2016). Informed consent was obtained from all participants before any samples were collected.

Measurement and evaluation

Prior to treatment initiation, patients underwent standard assessments of hematological and biochemical blood parameters, as well as a non-contrast-enhanced abdominal computed tomography (CT) scan. Routine laboratory analyses were performed using established techniques. These analyses encompassed leukocyte count, IL-6, CRP, fibrinogen, and erythrocyte sedimentation rate (ESR).

Interventions

Group A underwent ESWL treatment using the Dornier Compact Delta II device (Dornier Medizintechnik GmbH, München, Germany), which integrates ultrasonographic and fluoroscopic imaging, delivers high-energy density focused precisely on the concretion while safeguarding surrounding tissues from harm, and ensures minimal patient discomfort. The device facilitated precise positioning through an integrated isocentric ultrasound and X-ray system. The C-arm moved along two projections while core X-rays continuously passed through the center of the target zone. This equipment offered versatile imaging capabilities, including isocentric ultrasonography, allowing for precise targeting and real-time monitoring of the disintegration process.

The voltage of each shock wave was incrementally raised from an initial 12 kV to a final voltage of 19 kV. The procedure concluded either upon observing satisfactory fragmentation via fluoroscopy or after delivering 3500 shock waves at a pulse rate of 84-90/min. The ESWL procedure was carried out as an outpatient procedure with the application of standard analgesia. In some rare instances, mild analgo-sedation was administered. All patients treated with URS received a prophylactic dose of antibiotics. On the other hand, patients with asymptomatic bacteriuria treated with ESWL received antibiotic therapy but were excluded from the study due to potential bias.

In Group B, the treatment approach involved URS with contact ureterolith disintegration. The flexible ureterorenoscope utilized was the Olympus CYF Type V2 model (Olympus Corporation, Tokyo, Japan), equipped with a 2.2 mm working channel capable of accommodating common endoscopic probes and attachments. For the purpose of ureterolith disintegration, a holmium-based lithosurgical unit known as the LitHO 30W Holmium:YAG for lithotripsy (Ho:YAG) was employed (Quanta System S.p.A., Milan, Italy). This laser operated at a wavelength of 2100 mm, which proved highly effective for lithotripsy, cutting, removal, and achieving hemostasis, with notable absorption by fluids and biological tissues. The laser's penetration into surrounding tissue was minimal, typically reaching only 0.3-0.4 mm, causing negligible harm. The instrument automatically adjusted emission settings based on fiber diameter and the selected operating mode. In most cases, concretions measuring up to 1 cm in size could be clearly visualized and effectively fragmented using this technique.

Laboratory methods

The concentration of IL-6 in the subjects' serum was determined using the chemiluminescence immunoassay (CLIA) method on the automatic analyzer Immulite®1000 system (IMMULITE 1000 System, Siemens Medical). This analysis utilized the commercial kit Immulite 1000 IL-6 (Diagnostic Products Corporation), which included IL-6 test units, IL-6 substrate, IL-6 reagent, and IL-6 calibrators. The CLIA method is based on immuno-chemiluminescence, involving the selective binding of antibodies with markers to generate a measurable signal. The immulite system utilized antibody-coated plastic beads as the solid phase, alkaline phosphatase as the reagent, and chemiluminescent enzyme as the substrate. Each bead served as a reactor for immune reaction, incubation, washing, signal processing, and development. After a 60-minute incubation at 37°C, signal intensity was measured, allowing for the determination of IL-6 concentration in pg/mL based on a standard curve created by measuring an antigen of known concentration. The reference value range for IL-6 was 1.7-1000 pg/mL.

The BN II analyzer was used for measuring serum CRP concentration using particle-enhanced immunonephelometry. High-sensitivity CRP (DADE BEHRING) was utilized as a diagnostic tool, composed of polystyrene particles coated with CRP mouse monoclonal antibodies. The reference range for CRP using this method was 0 to 5 mg/L. Standard laboratory analyses included leukocyte count, fibrinogen levels, and ESR.

Statistical analysis

All data were analyzed using the statistical tool SPSS Statistics version 16 (SPSS Inc. Released 2007. SPSS for Windows, Version 16.0. Chicago, SPSS Inc.). The results were presented as mean standard deviation (SD), median (M), and interquartile range (IQR, 25th-75th percentiles). The distribution of variables was assessed using the Shapiro-Wilk test and the Kolmogorov-Smirnov test. For normally distributed independent variables, the Student's t-test was applied for comparative analysis, while the Mann-Whitney U test was used for non-normally distributed independent variables. For dependent variables lacking a normal distribution, either the Wilcoxon test or the Friedman test was employed based on the number of repeated measurements.

## Results

ESWL treatment group

IL-6 levels showed no significant difference between pre-intervention and the first post-intervention day (1.8 vs. 2.33 pg/mL, p=0.227). CRP levels increased significantly on the first post-intervention day (2.4 to 2.4 mg/dL, p<0.05) but decreased after six months (2.4 to 1.02 mg/dL, p<0.001) (Table 1).

**Table 1:** The effect of ESWL on hematological inflammatory parameters * p<0.05 in comparison to pre-intervention, # p<0.05 in comparison to first post-intervention day The independent t-test and paired t-test were used to compare the values

Variables	Pre-intervention	First post-intervention day	6 months after	p
Interleukin-6 (pg/mL)	1.8 (1.4–2.59)	2.33 (1.22–3.19)	-	0.227
C-reactive protein (mg/dL)	2.4 (1.0–6.1)	2.4 (0.8–6.9)^*^	1.9 (0.9–3)^*#^	<0.001
Leukocytes (x10e9)	7.62 (6.20–9.05)	7.24 (5.8–9.09)	7.0 (6.01–8.27)^*^	<0.05
Fibrinogen (g/L)	3.6 (2.9–4.3)	3.6 (3.1–4.3)	2.85 (2.25–3.1)^*#^	<0.001
Erythrocyte sedimentation rate (mm)	14 (7–25)	16 (11–32)^*^	9 (5–12)^*#^	<0.001

Leukocyte count decreased significantly after six months compared to pre-intervention (7.62 to 7.0 x10e9, p<0.05). Fibrinogen levels decreased significantly after six months compared to both pre-intervention and the first post-intervention day (3.6 to 2.85 g/L, p<0.05).

ESR levels increased significantly on the first post-intervention day compared to pre-intervention (14 to 16 mm, p<0.05) but decreased after six months compared to the first post-intervention day (16 to 9 mm, p<0.05) (Table 1).

URS treatment group

IL-6 levels significantly increased on the first post-intervention day compared to pre-intervention (2.9 to 7.1 pg/mL, p<0.001). CRP levels increased significantly on the first post-intervention day compared to pre-intervention (2.7 to 13.9 mg/dL, p<0.05) but decreased after six months compared to the first post-intervention day (13.9 to 2.9 mg/dL, p<0.05) (Table 2).

**Table 2:** The effect of ureteroscopy (URS) on hematological inflammatory parameters *p<0.05 in comparison to pre-intervention; # p<0.05 in comparison to first post-intervention day The independent t-test and paired t-test were used to compare the values

Variables	Pre-intervention	First post-intervention day	6 months after	p
Interleukin-6 (pg/mL)	2.9 (1.9–3.34)	7.1 (3.85–28.07)	-	<0.001
C-reactive protein (mg/dL)	2.7 (2.3–4.9)	13.9 (8.5–31.2)^*^	2.9 (1.9–4)^#^	<0.001
Leukocytes (x10e9)	7.24 (6.2–10.4)	8.9 (5.8–13.2)^*^	7.6 (6.9–8.3)^*#^	<0.05
Fibrinogen (g/L)	3.5 (3.1–4.3)	3.4 (3.1–5.1)^*^	2.8 (2.3–3.1)^*#^	<0.001
Erythrocyte sedimentation rate (mm)	16.5 (7–26.5)	24.5 (13–47)^*^	8 (5.2–10)^*#^	<0.001

Leukocyte count increased significantly on the first post-intervention day compared to pre-intervention (7.24 to 8.9 x10e9, p<0.05) and remained higher after six months compared to the first post-intervention day (8.9 to 7.6 x10e9, p<0.05). Fibrinogen levels decreased significantly after six months compared to both pre-intervention and the first post-intervention day (3.5 to 2.8 g/L, p<0.05).

ESR levels increased significantly on the first post-intervention day compared to pre-intervention (16.5 to 24.5 mm, p<0.05) but decreased after six months compared to the first post-intervention day (24.5 to 8 mm, p<0.05) (Table 2).

Comparative analysis of outcomes in the URS and ESWL groups

IL-6 levels were significantly higher in the URS group compared to the ESWL group both peri-interventionally and on the first post-intervention day (p<0.001). CRP levels were significantly higher in the URS group compared to the ESWL group on the first post-intervention day and after six months (p<0.001). The number of leukocytes and ESR were significantly higher in the URS group compared to the ESWL group on the first post-intervention day (p<0.001) (Table 3).

**Table 3:** Comparison of hematological inflammatory parameters of patients treated with ESWL and URS An independent t-test was used to compare the values ESWL: extracorporeal shock wave lithotripsy, URS: ureteroscopy

Variables	Pre-intervention			First post-intervention day			6 months after		
	ESWL group	URS group	p	ESWL group	URS group	p	ESWL group	URS group	p
Interleukin-6 (pg/mL)	1.8 (1.4–2.6)	2.9 (1.9–3.3)	<0.001	2.33 (1.2–3.1)	7.1 (3.7–28.4)	<0.001	-	-	-
C-reactive protein (mg/dL)	2.4 (1.1–6.3)	2.7 (2.3–4.9)	0.63	2.4 (0.8–6.9)	13.9 (8.1–31.2	<0.001	1.9 (0.9–3.0)	3.0 (1.9–4.02)	<0.001
Leukocytes (x10e9)	7.62 (5.98–9.06)	8.32 (6.2–10.4)	0.09	7.24 (5.8–9.0)	8.8 (5.8–13.2)	0.03	6.95±1.68	7.48±1.21	0.054
Fibrinogen (g/L)	3.6 (2.9–4.4)	3.4 (3.0–4,2)	0.19	3.6 (3.1–4.3)	3.3 (3.1–4.9)	0.62	2.85 (2.22–3.10)	2.80 (2.30–3.10)	0.70
Erythrocyte sedimentation rate (mm)	14 (7–25)	16.5 (7–26.5)	0.37	15.5 (11.0–32.0)	23.5 (13.0–47.0)	0.03	9.0 (5.0–12.0)	8.0 (5.0–10.0)	0.62

These findings suggest that URS treatment led to more pronounced changes in inflammatory parameters, particularly IL-6 and CRP, compared to ESWL treatment. However, it's important to note that some parameters improved or normalized over time in both groups, indicating potential recovery from the interventions.

## Discussion

The total number of cases and deaths attributed to urolithiasis has globally increased since 1990, necessitating a more intensive focus on the development of treatment modalities [1]. Although there have been notable advancements in the development of these techniques, there has also been the observation of significant adverse effects linked to an increase in inflammatory parameters.

While ESWL presents positive therapeutic outcomes, it also entails potential adverse effects resulting from the mechanical trauma induced by shock waves, often attributed to factors such as bubble cavitation and shear stress [3]. These occurrences are inherently associated with the inflammatory response within the tissues [4].

Despite being regarded as a minimally invasive procedure, the spectrum of complications subsequent to URS varies significantly, ranging between 9% and 25% [7]. Novel biological measures may hold the key to detecting issues such as bleeding, acute renal damage, and infection that could arise after both ESWL and URS. This is significant because, at present, there has been limited research on the pathophysiological impact of ESWL and ureterorenoscopy on clinical outcome indicators [8-10].

Our findings underscore the unique role of IL-6 as not just an indicator of tissue trauma invasiveness but also as a predictor for various post-procedural events, particularly in ESWL and URS contexts. However, the interpretation and utilization of IL-6 levels may vary based on individual patient factors, clinical context, and medical practices. In these procedures involving kidney or ureteral stone manipulation, IL-6 serves as an inflammation marker. Elevated IL-6 levels post-procedure can signify tissue damage and inflammation, potentially indicating infection risk, especially after URS. Monitoring IL-6 levels aids in assessing healing and recovery; decreasing IL-6 levels suggest reduced tissue damage, signifying positive recovery. This data can guide discharge decisions and further medical interventions. Additionally, IL-6 levels offer valuable insights for research, enabling investigations into correlations with patient outcomes, complications, and treatment efficacy in urinary stone management involving ESWL and URS [8-10].

Results of our study show that there was no significant difference in the levels of IL-6 between the pre-intervention and the first post-intervention day in patients with urolithiasis who underwent ESWL treatment. However, the IL-6 levels on the first post-intervention day increased compared to the pre-intervention measurements in patients with urolithiasis treated with URS treatment. Through comparative analysis, our research results demonstrated that IL-6 values were significantly higher in patients treated with URS treatment compared to those treated with ESWL treatment on the first post-intervention day. These findings align with Bantis et al.'s research, which also indicated a statistically significant increase in IL-6 levels 2 and 24 hours after URS treatment. Additionally, Bantis et al. monitored the trends in tumor necrosis factor-α (TNF-α) levels, which exhibited an upward trajectory in the post-intervention period, specifically one hour and 48 hours after surgery. The authors concluded that elevated pre-intervention levels of serum TNF-α and IL-6 may indicate a predisposition to post-intervention inflammation and infections following URS treatment [13].

In another study, Bantis et al. demonstrated a significant increase in serum IL-6 concentrations two hours, 24 hours, and 48 hours after ESWL. They also observed a significant rise in TNF-α values in the serum, both one hour and 24 hours after treatment [14].

Li et al. investigated the potential mechanism of ESWL-induced renal inflammatory damage in a study involving 48 rats. They examined the expression of TNF-α, intercellular adhesion molecule (ICAM)-1, and monocyte chemoattractant protein (MCP)-1 at 3 and 105 days after ESWL treatment. The researchers also monitored various markers, including malondialdehyde, β2-microglobulin, TNF-α, IL-6, and IL-18, in urine before and after treatment. Their findings revealed increased expression of ICAM-1 and TNF in the kidneys on the third day after ESWL, along with significantly elevated levels of TNF, IL-6, and IL-18 in urine compared to pre-intervention values. After 105 days, MCP-1 expression in the kidneys increased, and the levels of β2-microglobulin and IL-18 in the urine also rose significantly [15].

Despite the advantages and effects of ESWL treatment, it is important to consider its potential side effects, such as adverse effects extending from the papilla to the outer cortex. Goktas et al. examined levels of IL-6, IL-1a, and TNF-α in patients with urolithiasis. They discovered that 24 hours after treatment, IL-6 in the urine increased significantly, in contrast to IL-1a, which increased after 14 days of ESWL treatment, while IL-6 decreased to a level comparable to the control group. The urinary component of TNF-α was not detected either before or after ESWL treatment [16].

Urolithiasis is known to cause severe pain and muscle contractions. Moreover, urinary stones can damage the endothelium, triggering an inflammatory response. IL-6 secretion in urolithiasis can occur through two different mechanisms. Endothelial damage may increase the risk of infection, while muscle contractions alone are insufficient to produce this effect. Therefore, it is crucial to identify factors and sources associated with increased IL-6 production [17].

Notably, glomerular injury is a potent stimulator of IL-6 production in tubular epithelial cells, representing one aspect of glomerular-tubular communication. Research has demonstrated that IL-6 can stimulate proximal tubular epithelial cells to produce collagen I, thereby accelerating tubulointerstitial fibrosis. Suppression of IL-6 expression in tubular epithelial cells prevented interstitial fibrosis and tubular atrophy, while chronic IL-6 administration exacerbated the fibrotic process. IL-6's involvement in renal innate cell injury, repair processes, and a range of renal diseases stemming from immune responses, metabolic factors, ischemia, and toxins has been clearly demonstrated [18].

To facilitate a comprehensive comparison of the two treatment methods for urolithiasis, our research also assessed treatment outcomes in patients who underwent ESWL and URS treatment based on inflammatory parameters. Sepsis as a complication after ESWL treatment was recorded in 1.5% of cases, while it occurred in 3.5% of cases following URS treatment [19].

In studies using serum markers of inflammation to predict the outcome of urinary stone cases, the number of leukocytes, neutrophils, CRP, and neutrophil-to-lymphocyte ratio were associated with the spontaneous passage of ureteral stones [20]. Corticosteroid drugs, known for their anti-inflammatory and anti-edematous effects, have been used in urolithiasis treatment because the presence of stones induces inflammatory mucosal reactions leading to edema. Previous studies have shown the positive effects of corticosteroids in facilitating stone passage or preventing related reactions [21].

In our study, the number of leukocytes did not significantly change during the follow-up period in patients treated with ESWL, whereas it increased significantly on the first post-intervention day and then decreased significantly after six months in patients with urolithiasis treated with URS. Our research findings indicated that sedimentation rates significantly increased on the first post-intervention day compared to pre-intervention measurements in both the ESWL and URS treatment groups. However, these rates decreased significantly after six months, reaching values significantly lower than those recorded on the first post-intervention day and compared to pre-intervention measurements.

CRP levels on the pre-intervention measurement day significantly increased on the first post-intervention day in patients with urolithiasis treated with both ESWL and URS treatment. However, after six months, these levels decreased significantly, reaching values significantly lower than those on the first post-intervention day.

Furthermore, the fibrinogen levels decreased significantly after six months compared to pre-intervention values in both treatment groups. ESR exhibited a significant increase on the first postoperative day and then significantly decreased after six months in both groups.

CRP levels were significantly higher in patients with urolithiasis treated with URS treatment compared to those treated with ESWL on the first post-intervention day and after six months. Similarly, the number of leukocytes and ESR was significantly higher in patients treated with URS treatment than in those treated with ESWL on the first post-intervention day.

A study performed by Moyee et al. demonstrated a significant increase in the number of leukocytes 120 minutes after URS treatment, with this trend continuing significantly 240 minutes after the procedure. Neutrophils exhibited a similar pattern, with a decrease observed 30 minutes after treatment, followed by a significant increase 120 and 240 minutes after URS [22].

An increase in CRP concentration was recorded after ESWL, both in patients who developed AKI and those who did not undergo this treatment. The authors found that 72.22% of patients with AKI experienced a significant increase in serum CRP concentration (≥2 times higher than baseline) after ESWL, which was the only kidney injury observed within 24 hours.

Despite our findings, it's noteworthy that the average change in CRP concentration was nearly statistically similar in patients who developed AKI and those who did not [23]. Serum CRP, which is increasingly used as a marker to predict the spontaneous passage of stones from the distal part of the ureter, yielded interesting results in the study conducted by Hasan et al. They observed that 32.19% of patients with a CRP value ranging from 0.5 to 4.9 mg/dl experienced spontaneous stone passage, whereas a significant 89.7% of patients with a CRP value between 5 and 9.9 mg/dl exhibited spontaneous passage of concretions, highlighting the statistical significance of this difference [24].

Regarding fibrinogen, our findings align with the results of Wožniak et al.'s study, which reported no statistically significant changes in fibrinogen concentration in patients treated with ESWL before and after treatment [25]. In contrast, a study by Hughes et al. demonstrated a statistically significant decrease in fibrinogen concentration in patients treated with ESWL 120 minutes after the procedure compared to the baseline value. The authors suggest that the trend of decreasing fibrinogen concentrations after ESWL warrants further investigation to clarify the role of fibrinogen as a potentially modifiable risk factor for perioperative bleeding that may occur after ESWL [26].

This study included potential weaknesses and limitations due to a relatively small sample size and a short-term focus. A larger sample would provide more robust results. Additionally, the study primarily assesses the short-term impact of ESWL and URS on inflammatory parameters, focusing on the first postoperative day and six months afterward. It does not offer insights into longer-term effects or the potential for delayed complications. Furthermore, the study was conducted at a single center in Bosnia and Herzegovina. This may limit the generalizability of the findings to a broader population or other healthcare settings with different patient demographics and treatment protocols.

## Conclusions

Based on our research findings, it is evident that the concentration of IL-6 significantly increased in patients with urolithiasis treated with URS on the first postoperative day, whereas there was no significant change in IL-6 levels in patients treated with ESWL.

Our research findings suggest that monitoring IL-6 levels can offer valuable insights into the degree of inflammation and tissue damage during and following observed procedures, particularly among patients undergoing ureteroscopy, even within the initial days post-procedure.
